# Research progress on the lectin pathway of complement in IgA nephropathy

**DOI:** 10.3389/fimmu.2026.1757595

**Published:** 2026-02-27

**Authors:** Xiaoqing Yu, Hui Gao, Xifeng Sun

**Affiliations:** 1 The Second Clinical Medical College of Binzhou Medical University, Yantai, China; 2 Department of Urology, Zibo Central Hospital., Zibo, China; 3 Department of Nephrology, Zibo Central Hospital, Zibo, China

**Keywords:** C4d, complement system, crescent, IgA nephropathy (IgAN), lectin pathway (MBL), MASP

## Abstract

As an autoimmune disease, IgA nephropathy is pathologically characterized by the deposition of immunoglobulin A (IgA) in the glomerular mesangial area. Recent research has confirmed that the activation of the lectin pathway in the complement system may be related to the development and prognosis of IgA nephropathy (IgAN). These deposited immune complexes trigger the complement cascade, generating various inflammatory mediators that directly attack glomerular mesangial cells and promote mesangial matrix proliferation and crescent formation, ultimately leading to end stage renal disease. Therefore, an in-depth understanding of complement activation pathways not only provides potential non-invasive biomarkers (such as urinary complement components) for assessing disease activity and prognosis, more importantly, establishes a theoretical foundation for developing novel anti-complement targeted therapies. This holds promise for opening new directions in the personalized precision treatment of IgA nephropathy. This article reviews the research progress on the lectin pathway and its associated components in IgA nephropathy.

## 1 Introduction

As the predominant variant of primary glomerulonephritis worldwide, Immunoglobulin A nephropathy (IgAN) carries a substantial disease burden. First documented in 1968 by Berger and Hinglais, it continues to constitute a leading etiology of end-stage renal disease (ESRD) (1). The primary pathogenic factor of IgA nephropathy is the formation of abnormal IgA, which is galactose-deficient, whose increased “stickiness” leads to its deposition in the mesangial area. This event is central to triggering a sequence of histopathological alterations, notably mesangial cell proliferation and inflammation, the development of glomerulosclerosis, and associated podocytopathy (2, 3). It commonly occurs in young adults aged 20 to 40 (4). Current diagnosis is established through renal biopsy (2). It carries a high risk of progression, with approximately 20%-50% of patients advancing to end-stage renal disease within 20 years. Those who progress experience a reduced life expectancy of 6–10 years. Only 20% of patients maintain long-term kidney function. The incidence of IgAN shows significant geographic variation, with the highest rates observed in Asia (5). The clinical manifestations of IgAN are highly variable, ranging from acute kidney injury (AKI) to chronic kidney disease (CKD), macroscopic hematuria, isolated proteinuria, or urinary abnormalities. Younger patients are more likely to present with gross hematuria and abnormal urinary sediment. Obviously, in older subjects where the diagnosis is reached late, the clinical presentation at the time of diagnosis is chronic renal failure (1). Therefore, investigating its pathogenesis is particularly important for the treatment of IgAN.

## 2 Pathogenesis of IgAN

**Figure 1:**
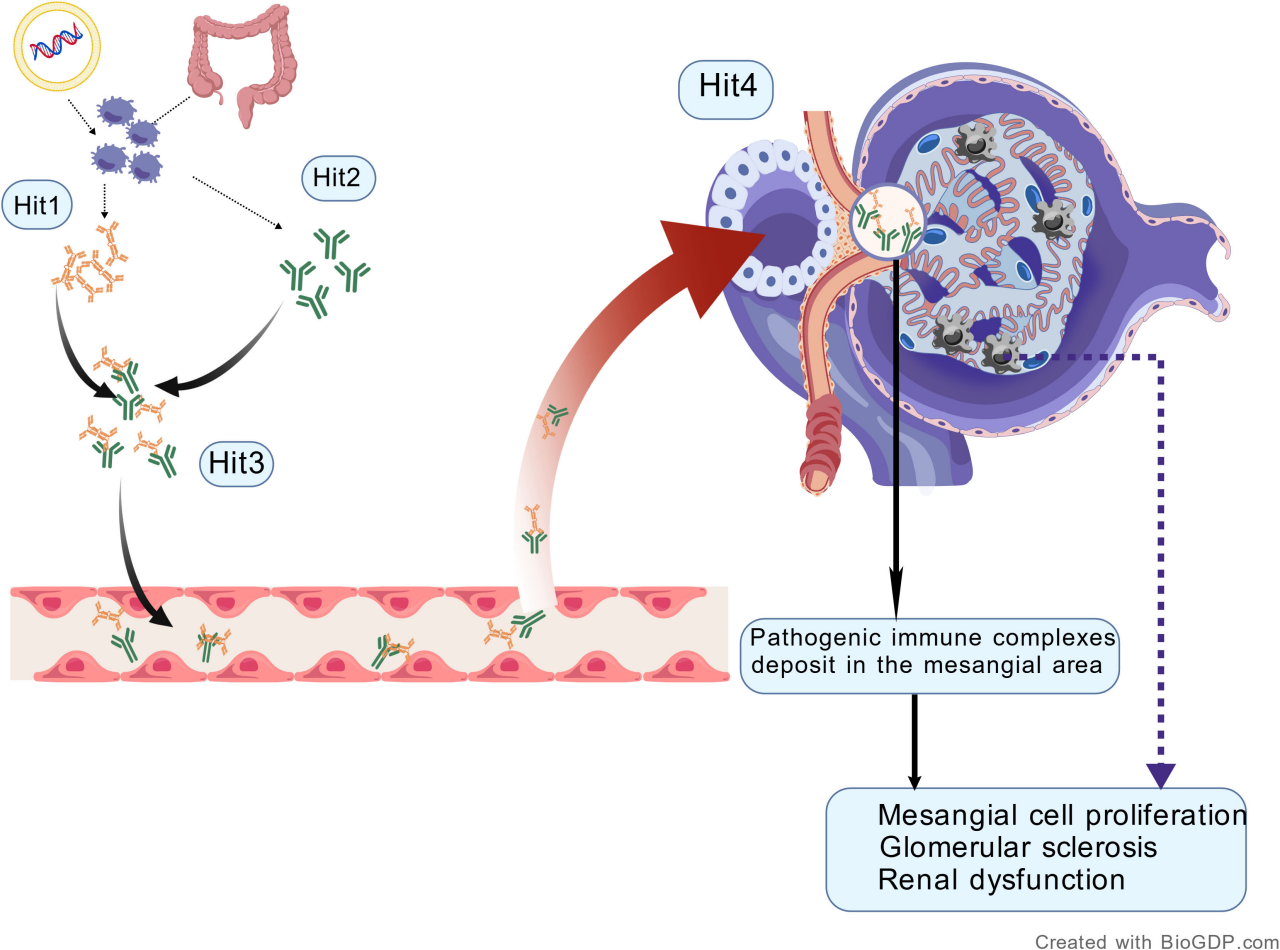
Four-hit hypothesis (Created with BioGDP.com).

Current research indicates that the pathogenesis of IgA nephropathy involves a “Four-hit hypothesis”. These steps include: abnormal glycosylation of IgA1, production of antibodies against galactose-deficient IgA1, formation of immune complexes through the binding of anti-glycan/glycopeptide antibodies to galactose-deficient IgA1, and the deposition of these complexes in the glomerular mesangium. The deposited immune complexes activate mesangial cells, leading to their proliferation, recruitment of inflammatory cells, and secretion of cytokines, which subsequently mediate inflammatory injury (6) (**Figure 1**).

Numerous studies indicate that renal histopathological changes and blood biomarkers suggest abnormal activation of the complement system. Activation of complement in serum or urine has been demonstrated to correlate with the severity and prognosis of IgAN (7). As a key component of innate immunity (8), it complement system includes the classical pathway (CP), the lectin pathway (LP), and the alternative pathway (AP). All three pathways converge into a common terminal pathway, ultimately forming the membrane attack complex (MAC), which causes cellular injury. Morito et al. detected glomerular deposition of MBL and MASP-1 in IgAN patients, which colocalized with C3b and C5b-9 deposition, but was independent of IgG, IgM, C1q, C4c, or properdin (9, 10), Renal biopsy further confirmed that complement activation in IgAN is predominantly via the alternative pathway (activated in 75–90% of patients) and the lectin pathway (activated in 17–25% of patients) (11, 12), while the classical pathway is not associated with IgAN (C1q is rarely observed) (13). A key finding from a 2001 follow-up study was the observation of glomerular mesangial co-deposition of MBL, MASP-1, and C4, which was present in more than half of the IgA nephropathy cohort. In these patients, IgA2 was found to colocalize with MBL and MASP-1 in the glomerular mesangium (14). Subsequently, other components of the lectin pathway, such as ficolin-2 deposition, were also identified in IgAN (11), More than 50% of IgAN patients showed renal deposition of MBL and/or C4d. The deposition of MBL, C4d, and L-ficolin often occurred simultaneously, collectively exacerbating mesangial proliferation, extracapillary proliferation, glomerulosclerosis, and interstitial infiltration. This led to a significant increase in proteinuria and ultimately poorer clinical outcomes (11, 15). In this review, we focus on discussing the relationship between the lectin pathway and the pathogenesis of IgAN, along with recent research advances.

## 3 The lectin pathway in IgAN

### 3.1 Overview of the lectin pathway

**Figure 2:**
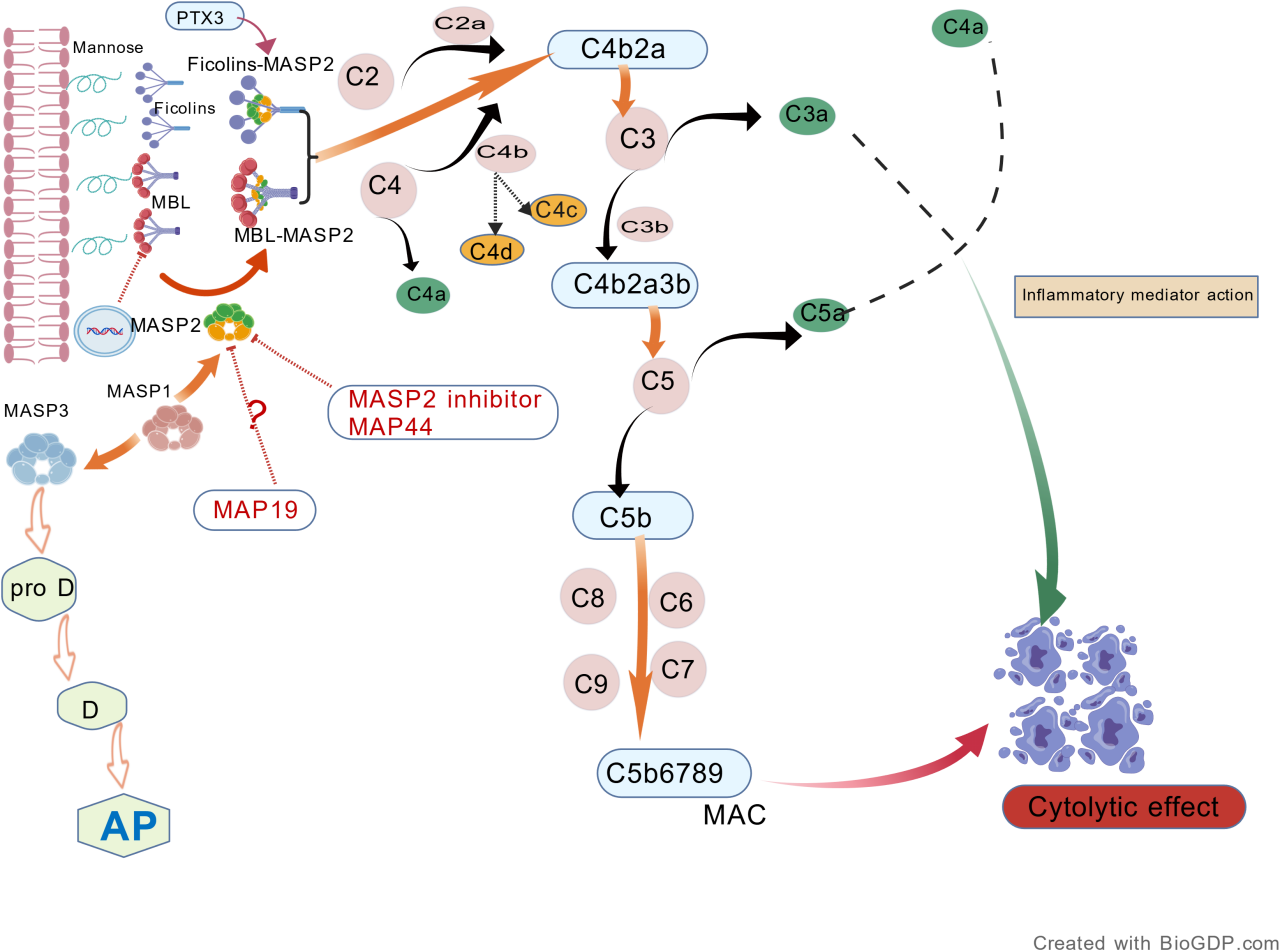
Schematic diagram of the lectin pathway (Created with BioGDP.com).

The pattern recognition molecules of the lectin pathway include mannan-binding lectin (MBL), L-ficolin, M-ficolin, H-ficolin, collectin liver 1 (CL-L1), and collectin kidney 1 (CL-K1). Upon recognition of pathogen-associated molecular patterns (PAMPs) or damage-associated molecular patterns (DAMPs), MBL triggers complement activation through the associated serine proteases MASP-1, MASP-2, and MASP-3. The activation of this pathway culminates in the formation of the membrane attack complex (MAC): the sequence begins with C3 convertase (C4b2a) assembly, proceeds through C5 cleavage to generate C5b in the terminal pathway, and concludes with the C5b-9 complex assembly, which mediates cytolytic cell injury (16). Meanwhile, the cleavage products of C3 and C5—C3a and C5a—act as pro-inflammatory factors that initiate inflammatory responses, further exacerbating tissue injury. During complement activation, C4b is further processed into C4d. C4d deposition, a degradation product of complement activation, reflects the activation of both the classical and lectin pathways (17), In IgAN, the absence of C1q deposition supports the conclusion that C4d formation is associated with the lectin pathway. (**Figure 2**).

It is important to note that during the cascade activation of the complement lectin pathway, the C3 convertase (C4b2a) further cleaves the central protein of the complement system, C3, forming C3a and C3b. C3b covalently attaches to the surfaces of nearby pathogens, apoptotic cells, or damaged tissues (18). Surface-bound C3b plays a key role in amplifying the complement cascade. It binds to factor B, which, under the catalytic cleavage of factor D, is cleaved into the inactive fragment Ba and the enzymatically active fragment Bb. Bb further assembles with C3b to form the C3 convertase of the alternative pathway (C3bBb). C3bBb can more efficiently cleave C3, continuously generating new C3b, and amplify complement activation through a positive feedback loop (19). Thus, the generation of C3b serves as a critical bridge connecting the lectin pathway with the subsequent powerful amplification loop of the alternative pathway (20). In a restricted group of subjects, it seems to participate along with the alternative pathway in dysregulated complement activation, and it may be related to the prognosis of the disease (21, 22).

### 3.2 Molecules involved in lectin pathway

#### 3.2.1 MBL

Mannose-binding lectin (MBL) is the core molecule initiating the lectin complement pathway. The binding between MBL and IgA is calcium-dependent and can be blocked by pre-incubating IgA with specific carbohydrates (23). This indicates that the interaction occurs between the carbohydrate recognition domain of MBL and specific glycosylated moieties on polymeric IgA (24). MBL, a member of the collectin subclass within the C-type lectin superfamily, recognizes and binds to Gd-IgA1 immune complexes deposited in the mesangial area. Furthermore, IgA2 deposition in the glomerular mesangium can activate the lectin pathway (14). MBL exists in multimeric forms, with each subunit consisting of a collagen-like domain (CLD) and a carbohydrate recognition domain (CRD) (23). The collagen-like domain mediates downstream signaling, while the carbohydrate recognition domain specifically recognizes high-mannose type glycans, thereby triggering the MBL-associated serine protease (MASP)-mediated complement cascade (25).

##### 3.2.1.1 MBL levels

It is abnormal MBL levels, encompassing both deficiencies and elevations, that constitute a risk factor for IgAN. As a key component of the innate immune system’s “first line of defense,” MBL recognizes and clears pathogens through opsonization, which is essential for resisting infections. Evidence indicates that low serum MBL levels or MBL2 genotypes associated with reduced MBL production are linked to susceptibility to various infections (26), For example, in infants and young children whose adaptive immune systems are not yet fully developed, MBL deficiency is a significant cause of recurrent infections (27, 28).

Studies have shown that MBL knockout mice exhibit higher susceptibility to *Staphylococcus aureus* infection compared to normal mice (25). Similarly, reduced levels of MBL have been implicated as a contributing factor in the progression of IgAN. Further analysis of urine from IgAN patients via gel electrophoresis revealed significant downregulation of MBL in those with progressive disease (29).In contrast, a study by Ohsawa et al. suggested that IgAN patients with MBL deficiency showed a trend toward improved proteinuria and renal function (30). Similarly, research by Roos A indicated that severe histopathological damage in IgAN patients was associated with glomerular deposition of MBL and L-ficolin (11). A notable limitation of that study is the lack of circulatory systemic MBL level measurements, which prevented any evaluation of a correlation with glomerular MBL deposition (11). These seemingly contradictory findings may be attributed to factors such as relatively small sample sizes, baseline heterogeneity among study populations (including infection status, treatment regimens, and comorbidities), wide variations in sample size (ranging from 6 to 749 subjects), and short follow-up durations, all of which could introduce bias. Such discrepancies may also reflect the high heterogeneity inherent in IgAN.

Subsequently, Guo et al. measured MBL levels in 749 biopsy-proven IgAN patients and 489 healthy controls. They found that, compared to the normal range (100–3540 ng/ml), both MBL deficiency and MBL overexpression (>3540 ng/ml) were associated with more severe proteinuria (P < 0.01) and higher Oxford C scores (P = 0.05) (26). These findings imply a pathogenic role for MBL dysregulation in IgAN, where both insufficiency and excess are associated with deteriorating renal function. Given that IgAN is closely associated with infections—often presenting with macroscopic hematuria following infections—and multiple studies have confirmed the presence of renal inflammation in IgAN patients (31), infections are known to trigger or exacerbate the disease. It can be hypothesized that when MBL is deficient, limited complement activation may fail to control infections, leading to disease activity. On the other hand, infections can induce active renal lesions, resulting in excessive complement activation and subsequent inflammatory injury. These two mechanisms may contribute to IgAN pathogenesis in different ways, with MBL levels potentially playing a dynamic role depending on the disease stage and genetic background of the patient. Therefore, future therapeutic strategies for IgAN should not focus solely on MBL supplementation or inhibition. Instead, they should incorporate individualized MBL level assessment to enable precise subtyping and personalized management.

##### 3.2.1.2 MBL gene polymorphisms

Recent advances in genetics and molecular biology suggest that gene polymorphisms may influence the progression of IgAN. Gorgi Y et al. confirmed an association between MBL polymorphisms and severe sporadic IgAN (32). Humans possess two MBL genes, *MBL1*and *MBL2*, but only *MBL2* is functional and encodes the active protein (23). Serum MBL levels are influenced by genetic variations in the promoter and coding regions of *MBL2* (NG_008196.1), particularly involving six single nucleotide polymorphisms (SNPs) in exon 1 and the promoter region (33). Functionally, these SNPs disrupt the formation of MBL oligomers, leading to MBL deficiency and reduced ability to recognize pathogens and activate complement (34). Gong et al. conducted the inaugural investigation into the association between MBL gene polymorphisms and the heterogeneity of immune deposition in IgAN. The researchers identified specific MBL gene defects as a cause of diminished serum MBL levels and demonstrated a correlation between these low levels and abnormal deposition patterns in a subpopulation of IgAN patients, thereby linking genetic predisposition to pathological outcomes. Specifically, the GAC variant at codon 54 was significantly associated with an “abundant glomerular immune deposition pattern” (AGM type) in IgAN (35, 36). Similarly, Shih et al. demonstrated that insufficient MBL activation due to a codon 54 mutation in the promoter region was linked to poor prognosis in progressive IgAN (29). Yan et al.’s MBL2 gene sequencing study identified 37 relevant variants. They reported that the rs1800450-AA genotype, which results in absent MBL expression in serum and renal tissue, is consequently associated with more severe tubulointerstitial damage and a 12.06 times elevated risk of end-stage renal disease. Notably, among the four common haplotypes, only the GCA haplotype correlated with disease progression (37). However, a study in China suggested a trend toward a lower frequency of the O allele (which leads to reduced functional MBL levels) in IgAN patients, implying that low MBL production may have a protective effect against the disease (26). Genetic variations and widespread polymorphisms in the *MBL2*gene contribute to significant differences in circulating MBL levels across populations (26). Therefore, the impact of *MBL2*gene polymorphisms on IgA nephropathy exhibits a complex dual nature. The central controversy lies in the fact that low MBL expression may exert either protective or detrimental effects depending on the clinical context, and the specific mechanisms involved require further in-depth investigation.

#### 3.2.2 Other pattern recognition molecules

##### 3.2.2.1 Ficolin

Humans express three types of ficolins—Ficolin-1 (M-ficolin), Ficolin-2 (L-ficolin), and Ficolin-3 (H-ficolin)—which can initiate the lectin pathway of complement activation by associating with MASPs or promote opsonophagocytosis to limit infections and coordinate subsequent adaptive immune responses (38). Both MBL and ficolins activate MASP-2 to cleave C4 and C2, forming the classical C3 convertase (C4b2a). However, the C3 convertases generated may differ in stability and activity (39). Ficolins exhibit a stronger ability to recognize endogenous “altered self” molecules, such as abnormal glycans exposed on apoptotic/necrotic cells and aberrantly glycosylated serum proteins. Their unique fibrinogen-like domains make them particularly involved in the pathogenesis of autoimmune diseases (39, 40). In IgAN patients, galactose-deficient IgA1 (Gd-IgA1) exposes abnormal N-acetylgalactosamine epitopes, which can be effectively recognized by ficolins (especially FCN2 (Ficolin-2) and FCN3 (Ficolin-3)) (3, 41), triggering lectin pathway activation. In a European cohort of 60 IgAN patients, glomerular MBL staining colocalized with Ficolin-1, MASPs, and C4d deposits, which correlated with significant histological injury (11). Similarly, Roos et al. first reported in 2005 that glomerular Ficolin-2 staining correlates with the severity of IgA nephropathy, but not with circulating Ficolin-2 levels (11). In 2017, Nicholas et al. found that plasma levels of both Ficolin-1 and Ficolin-2 were elevated in patients with progressive IgAN compared to controls. Ficolin-1 levels were negatively correlated with estimated glomerular filtration rate (eGFR) and appeared to be influenced by both disease progression and eGFR (16). The functional activity of Ficolin-2 is also affected by genetic variations in the *FCN2*gene (NG_011649.1) (42). A study by Yan Ouyang et al. suggested that the rs7851696 SNP in the *FCN2*gene was associated with an increased risk of ESRD and Ficolin-2 levels in univariate Cox analysis. However, this association was not significant in multivariate analysis, indicating that the effect of the *FCN2*SNP on disease progression may not be independent and could be influenced by other factors (37). Nevertheless, it may still represent a potential risk factor for IgAN progression. In a proteomic analysis of serum from 60 IgAN patients and 43 healthy controls, Dandan Xue et al. observed upregulation of Ficolin-3 expression across different stages of IgAN (43). In a cholangiocarcinoma study, Zhang et al. demonstrated that FCN3 overexpression suppresses tumor cell proliferation and migration, suggesting a mechanism where FCN3 binding to MASP2 enhances complement-mediated cytotoxicity, thereby revealing a potential pathway for FCN3-modulated tissue injury via complement activation (44), although this mechanism has not yet been directly validated in IgAN models. Overall, the specific roles and mechanisms of different ficolin subtypes (Ficolin-1, Ficolin-2, and Ficolin -3) in the development and progression of IgAN remain inadequately studied and require further investigation.

##### 3.2.2.2 CL-L1and CL-K1

Hepatic collectin-1 (CL-L1) and renal collectin-1 (CL-K1), as key members of the collectin family, have been demonstrated to play distinct roles in the pathological process of IgA nephropathy (IgAN). Their mechanism of action involves forming functional complexes with MBL-associated serine proteases (MASPs) to jointly drive the cascade activation of the complement system (45). Primarily expressed in the adrenal glands, kidneys, and liver, CL-K1 exhibits a binding affinity for L-ficolin and D-mannose. This specific carbohydrate recognition enables its interaction with a spectrum of microorganisms and the serine proteases MASP-1/MASP-3, thereby initiating complement activation and facilitating the opsonophagocytosis of pathogens (46). CL-K1 exhibits predominant expression in renal tissue. It functions as a key mediator by binding to mesangial IgA1, thereby initiating lectin pathway activation and C3 deposition (47). Furthermore, locally produced CL-K1 within the kidney actively exacerbates the pathological progression of both glomerular and tubulointerstitial fibrosis (48). Consequently, inhibiting MASP-2, may confer a protective effect on renal structures in IgAN patients (47). Conjugation of CL-L1 and CL-K1 *in vivo* yields the CL-LK heterocomplex, a molecular scaffold that mounts a robust immune response via the lectin pathway. This complex exhibits calcium-dependent stabilization with both MASP-1/3 and MASP-2. Reconstitution experiments demonstrate that ligand recognition by CL-LK (targeting specific carbohydrates or negative charges) promotes engagement of circulating MASP-2, culminating in C4 conversion and complement cascade initiation (49). However, the specific roles of CL-K1 and CL-LK in the lectin pathway within the pathogenesis of IgAN remain poorly characterized. Further research is needed to elucidate their molecular mechanisms in this context.

#### 3.2.3 Other related molecules: pentraxin 3

Pentraxin-3 (PTX-3) is a pattern recognition molecule that mediates innate immune responses. It can be produced by various extrahepatic tissues and blood cells under stimulation from inflammation and infection. Also known as TNF-α-induced protein 14, PTX-3 functionally activates the complement system and acts as a chemokine for a range of inflammatory cells (50, 51). In IgA nephropathy, mesangial cells have been confirmed to both produce and activate PTX-3 (52). For instance, early research by Benedetta et al. showed strong positive staining for PTX-3 in the glomerular mesangial areas of IgAN patients. *In vitro* experiments further confirmed that stimulation of cultured mesangial cells with IgA or TNF, a cytokine associated with IgAN, increases PTX-3 synthesis, indicating a specific PTX-3 induction pathway in mesangial cells that may be related to the pathogenesis of IgAN (52). The complement alternative pathway is known to play an essential role in the pathogenesis of IgAN (53). PTX-3 acts as a positive regulator of this pathway by stabilizing the C5 convertase (C3bBb3b) (54), thereby amplifying its positive feedback effect (55).

Involvement of PTX-3 in the complement lectin pathway has also been reported. For example, Guo et al. observed a trend of colocalization between PTX-3 and MBL in the renal mesangium of patients with higher MBL levels (26). A recent cohort study involving 96 patients (65 IgAN, 31 IgAVN ( IgA Vasculitis Nephritis )) found that patients with detectable PTX-3 and MBL in their urine more frequently showed mesangial cell proliferation, endocapillary hypercellularity, and crescent formation on renal biopsy (56). Furthermore, studies indicate that PTX-3 can interact with ficolin-1 and ficolin-2. The interaction with ficolin-2 enhances the complement deposition mediated by the latter, suggesting this interaction may have significant physiological implications (57). Research by Ma Yingjie et al. on the interaction between MBL and PTX-3 indicates that MBL binds to PTX-3 partly via its collagen-like domain. This binding forms an MBL-PTX-3 complex, which can subsequently recruit C1q, thereby establishing a functional link between the lectin pathway and the classical pathway of complement activation (58). However, the specific molecular mechanisms by which the MBL-PTX-3 axis promotes the progression of IgAN remain to be further elucidated.

#### 3.2.4 Core functions of MASP proteases

Four MASPs have been identified: MASP-1, MASP-2, MASP-3, and sMAP/MAP19. The binding site for MASPs and MAPs is located within the collagen-like domain of MBL (25, 59). MASP-1 and MASP-2 are encoded by distinct genes and contain serine protease domains (60). In contrast, MASP-3 and sMAP are alternative splicing products of the *MASP1*and *MASP2*genes. It is hypothesized that these two MASPs may act as regulators of the lectin pathway. MBL and MASPs circulate in the bloodstream as preformed complexes (25, 61).

##### 3.2.4.1 MASP-2

As the primary activator of the lectin pathway of complement, MASP-2 plays multiple roles in IgA nephropathy (IgAN). First, it binds to and is activated by MBL, which recognizes mannose residues on pathogens, thereby initiating the complement cascade and contributing to immune defense. Second, MASP-2, a key protein in the lectin pathway, is present not only in the blood but also in urine. Notably, MASP-2 in urinary extracellular vesicles (UEVs) has emerged as a potential non-invasive biomarker for IgAN. In a cohort of 38 IgAN patients and 17 controls, serum MASP-2 levels showed no significant intergroup difference. However, MASP-2 levels in urinary extracellular vesicles (UEVs) were markedly elevated in the IgAN group and demonstrated specific correlations: positive with urinary microalbumin and blood urea nitrogen, and inverse with serum albumin (62). Another study of 143 IgAN patients and 60 controls found that patients with low (<224.22 ng/ml) or high (>2540.50 ng/ml) serum MASP-2 concentrations had significantly lower renal survival rates (P < 0.05). Serum MASP-2 may thus serve as a novel biomarker, providing reliable support for the diagnosis, treatment, and prognosis of IgAN. Beyond cleaving C2 and C4, MASP-2 has additional functions: it can activate coagulation factor XII, triggering the coagulation cascade, converting prothrombin to thrombin, promoting fibrinogen turnover and clot formation. This may lead to increased coagulation and potentially contribute to thrombotic microangiopathy (TMA) (63). Chua et al. examined 42 kidney biopsy specimens from different patient populations and detected C4d deposition in 88.1% of TMA cases, which was associated with poor prognosis (64). Complement activation products C3a and C5a further enhance platelet aggregation and coagulation factor release (65), thereby forming a vicious cycle. In IgAN, since circulating macromolecular IgA1 primarily interacts with the glomerular capillary wall, endothelial injury—combined with immune complex deposition and lectin pathway activation—promotes TMA development (57). Consequently, MASP2 exacerbates renal dysfunction by dual mechanisms involving complement activation and coagulation cascade-mediated thrombosis in IgAN.

##### 3.2.4.2 MASP-1/MASP-3

MASP-3 differs from MASP-1 only in its serine protease (SP) domain (66). MASP-1 appears to cleave C2 and C3 but not C4 (60). Initially, MASP-1 contributes to lectin pathway activation. While MASP-2 was traditionally regarded as the autonomous activator of the lectin pathway, with MASP-1 playing only an auxiliary role, studies in MASP1/3 knockout mice revealed complete loss of serum MBL, MASP-1, and MASP-3, along with abolished lectin pathway activity. However, supplementation with recombinant MASP-1 restored lectin pathway function (67). Additional evidence confirms MASP-1 as a complement-activating enzyme, whereas MASP-2 serves as the autonomous activator of the lectin pathway. Following pattern recognition molecule (PRM) binding to activating surfaces, MASP-1 undergoes autoactivation first, and activated MASP-1 becomes the exclusive activator of MASP-2. Kinetic analyses further support the central role of MASP-1 in lectin pathway initiation (68). Regarding MASP-3, when it binds to pattern recognition molecules (PRMs), it can displace MASP-2 and MASP-1, thereby inhibiting complement activation. Thus, low levels of MASP-3 may be associated with increased complement activation (69). Studies have shown that MASP-3 levels are inversely correlated with the severity of IgA nephropathy. It has been confirmed that in resting human blood, MASP-3 serves as the exclusive physiological activator of pro-factor D (70). Through specific inhibition experiments, MASP-3 inhibitors were found to completely block the cleavage of pro-factor D, whereas inhibitors of MASP-1 or MASP-2 had no such effect (66). This finding links the lectin pathway to the alternative pathway to some extent and may elucidate the connection between these two complement activation pathways in the pathogenesis of IgAN. PCSK6 has been identified as the primary activator of MASP-3 (39, 64). MASP-3 deficiency may contribute to complement dysregulation, representing a potential underlying mechanism for enhanced activation. Some researchers hypothesize that abnormal activation of the alternative pathway during IgAN progression leads to substantial consumption of MASP-3, resulting in decreased circulating MASP-3 levels. However, the relationship between lectin pathway activation and MASP-3 levels, as well as the underlying mechanisms in IgAN, remain unclear.

##### 3.2.4.3 MAP19/MAP44

Pattern recognition molecules can also bind to non-enzymatic subunits—MBL-associated proteins MAp19 and MAP44 (16). MAp19/sMAP is a truncated gene product of MASP2, while MAP44 (also termed MAP-1) is a splice variant of the MASP1 gene, forming a 44 kDa protein that lacks the serine protease (SP) domain. Studies indicate that both proteins can regulate lectin pathway activity by competitively binding to ficolins and inhibiting the activation of mannan-binding lectin-associated serine proteases (MASPs) (40, 71, 72). However, Degn et al. developed MAp19-specific monoclonal antibodies and quantitatively measured MAp19, finding that it does not compete with MASP-2 for binding to MBL and therefore does not inhibit complement activation (73). Subsequently, in a cohort comprising 125 IgA nephropathy patients and 211 controls, elevated plasma levels of MAp19 were observed in IgAN patients and were inversely correlated with eGFR. Patients with biopsy evidence of mesangial hypercellularity, tubular atrophy, and segmental sclerosis showed higher plasma MAp19 levels (16). Thus, the significance and physiological relevance of elevated MAp19 levels in the progression of IgAN remain unclear and may be associated with different renal pathological changes. The role of MAP44 in the pathogenesis of IgAN also requires further investigation.

We have summarized the research status of different lectin components in IgAN disease and further analyzed the strength and weakness of the existing evidence. See **Table 1** for details.

**Table 1:** Research status of different lectin components in gAN disease.

Components	Codinggene location	Structure or function	Research core in IgAN	Evidence analysis	References
MBL	MBL/10q11.2-q12	Contains CLD and CRD domains;CRD can specifically recognize oligo-high mannose-type glycans	Co-deposits with IgA1, serum levels have a non-linear impact on prognosis, and gene polymorphism is a risk factor for the prognosis	Clinical and genetic evidence chain is complete; with a clear association.	(26, 29, 30, 37)
MASP-1	MASP1/3p27	Cleave C2 and C3	After self-activation, it activates MASP-2. .	Lack of large-sample prognostic association studies	(67, 68)
MASP-2	MASP2/1q36	Main activator of the lectin pathway; Activates coagulation factor XII	Mediates the complement cascade, induces TMA; MASP2 in serum and UEVs can predict the prognosis.	Supported by mechanisms and clinical trials with sufficient evidence	(62–64)
MASP-3	MASP1/3p27	Activate the precursor factor D; Synergistic with alternative pathways	MASP-3 levels are negatively correlated with the severity of IgA nephropathy, possibly due to abnormal activation of the alternative pathway.	The specific mechanism is unclear	(66, 68)
MAP44	MASP1/3p27	Contain a double CUB domain, no SP domain; Associate with ficolin	Negatively regulates the lectin pathway.	No specific research evidence for IgAN	(72)
MAP19	MASP2/1q36	Contains CUB1 and EGF domains; Not compete with MASP-2 for binding to MBL	The plasma MAP19 level increases with the aggravation of IgAN pathological damage.	The significance and physiological relevance of elevated MAp19 levels remain unclear.	(26, 43)
M-ficolin(ficolin-1)	FCN1/9q34.3	Associate with MASP	Co-localize with ficolin-1, MASPs, and C4d deposits, which is influenced by various factors.	It is only confirmed that there is deposition, and the mechanism is unknown.	(21)
L-ficolin(ficolin-2)	FCN2/9P34.3	Synthesized by the liver; Containing a fibrinogen-like domain; Expression is regulated by the FCN2 gene; Associate with the MASP activation pathway.	The role of FCN2 gene polymorphism in IgAN is unclear, and it may affect its expression (no significance in multivariate analysis)	There is no support from IgAN-specific research.	(11, 42)
H-ficolin(ficolin-3)	FCN3/1p5.3	Contains a fibrinogen-like domain; Preferentially recognizes acetylated groups; Associate with the MASP activation pathway	Deposits can be detected in IgAN kidney tissues, participating in the initiation of pathways.	The mechanism is unknown.	(43, 44)
CL-L1/CL-K1	colec10,colec11	Form CL-LK oligomers, promote the recruitment of MASP-2.	Possessing activation ability, Inferred to be a collaborative pathogenesis	Only speculation about structural functions, with no direct evidence	(49)
PTX3		Produced by extrahepatic tissues and blood cells; Activate the complement system and serve as a chemokine for inflammatory cells.	PTX3 and MBL are co-deposited, synergistic effect with Ficolin-2.	It is only confirmed that there is deposition, and the mechanism is unknown.	(26, 56–58)

## 4 The clinical and pathological relevance of the lectin pathway in IgA nephropathy

### 4.1 Renal tissue deposition and complement activity

Complement activation plays a critical role in the progression of IgAN, which may be initiated either systemically in the circulation or locally on mesangial-deposited IgA1-containing immune complexes (74). In both most MBL-positive and MBL-negative patients, glomerular C3 deposition has been observed, which differs from lectin pathway deposition seen only in a few patients with specific clinicopathological features. The activation of the alternative pathway is more widespread, and the abundance of C3 deposition in the mesangium and capillary walls is associated with the severity and progression of IgAN (24). This pathway also involves members of the complement factor H-related proteins (CFHRs) family. Current evidence indicates that the co-deposition of CFHRs and C3 is a marker of disease activity (75), directly promoting disease progression, negatively impacting the MEST score of renal pathology, and exacerbating renal injury. However, it is worth mentioning that the local synthesis of multiple factors—especially C3—occurs under physiological conditions and is further upregulated by pathological stimuli. While this contributes to pathogenesis, it remains to be fully elucidated whether the deposition of this complement factor directly causes damage or represents a self-repair response by the kidneys. Current research has not yet fully elucidated its mechanism. Moreover, given that the complement deposition observed in biopsy “snapshots” cannot reflect the dynamic changes of complement deposition nor distinguish the source of complement, the scientific community should begin to critically evaluate the specific role of this local product.

C4 is a key component of the complement system. It is a glycoprotein composed of three chains—alpha, beta, and gamma—linked by disulfide bonds (64, 76, 77). In the lectin pathway, MASP-2 cleaves complement component C4 at a single site within the α-chain’s amino-terminal region, generating C4a and C4b (77). C4b combines with C2a to form C4b2a, which initiates the complement cascade amplification and mediates inflammatory injury in the mesangial area as well as cell activation. The free C4b that does not participate in convertase assembly undergoes a conformational change to become iC4b, and is then further processed under the combined action of protease factor I and cofactor CD46 (78, 79). It is further cleaved into C4d linked by a thioester bond and soluble C4c. During this process, the generation of C4d depends on local supply of the C4 precursor and activation of specific enzymatic systems. C4d covalently and stably binds to the capillary wall or mesangial matrix at the activation site through its thioester bond. Ultimately, it results in the synchronous hyperactivity of the pathological cascade mediated by C4b2a and the secondary accumulation of C4d. As a key marker of complement activation, the significance and initiating pathway of mesangial C4d deposition have drawn considerable attention (74). Multiple studies have confirmed that glomerular C4d deposition strongly correlates with disease severity and poor prognosis in IgAN (80, 81). Espinosa et al. demonstrated that patients with positive mesangial C4d staining exhibited more severe renal impairment, more advanced glomerulosclerosis, and a lower 10-year renal survival rate (82). A subsequent large cohort study further established C4d deposition as an independent risk factor for progression to end-stage renal disease in IgAN (15). Sahin et al. also observed that C4d-positive patients exhibited significantly more severe renal pathological damage and a markedly higher rate of progression to ESRD (83). Heybeli et al. further indicated that glomerular C4d deposition in IgAN patients was associated with more severe proteinuria, lower eGFR, more prominent endocapillary hypercellularity, and a significant correlation with the T2 lesion score in the Oxford Classification (84). A meta-analysis by Jiang et al., which included 1,251 IgAN patients from different regions, demonstrated that patients with C4d deposition had lower eGFR, higher urine protein-to-creatinine ratio or 24-hour urine protein excretion, and an increased risk of hypertension. Furthermore, glomerular C4d deposition correlated with higher Oxford Classification scores (M, E, S, T), and these patients were more frequently treated with renin-angiotensin system blockers and immunosuppressive agents (85). A recent study also confirmed that glomerular C4d deposition is associated with M1, E1, S1, and T1/2 lesions in the Oxford Classification, and correlates with class C1–C2 pathology, further underscoring its potential value as a prognostic biomarker in IgAN (86). In routine renal biopsy practice, C4d staining is a relatively low-cost, easily performable assay with straightforward interpretation and high specificity (15). Currently, C4d staining is routinely used in the diagnosis of antibody-mediated rejection in renal transplantation, providing a practical foundation for its application in the clinical evaluation of IgAN. However, it should be acknowledged that only a single domestic study has documented the association between focal segmental C4d deposition along glomerular capillary walls (as opposed to mesangial deposition) and a poorer prognosis (87, 88). The mechanism underlying focal C4d deposition along glomerular capillaries in IgAN may be associated with local complement synthesis amplification and aberrant activation of the intracellular complement system (the complosome) (89). Specifically, activated complosomes in endothelial cells and podocytes can enhance intracellular inflammatory signaling, upregulate cell-surface complement receptor expression, and thereby promote the focal anchoring of C4d along the capillary wall, forming a pathological pattern of “intracellular-extracellular complement synergy. This alteration in the deposition pattern is not only correlated with the severity of renal pathological damage but also influences the characteristics observed by immunofluorescence (IF) and is closely related to the patient’s response to treatment. At present, there is insufficient focus on the implications of C4d deposition at different anatomical sites. Moreover, the prognostic significance of C4d deposition still lacks support from robust prospective randomized controlled trials. Additionally, standardized criteria for interpreting positive C4d staining and consistent methodological guidelines have not been established nationwide. These gaps highlight key directions for future research.

### 4.2 Pathological changes

#### 4.2.1 Formation of crescents

The clinical course of IgAN varies widely, ranging from isolated hematuria to rapidly progressive renal failure, while pathological findings span from mild mesangial hyperplasia to diffuse crescent formation. Complement activation—particularly involving the alternative and lectin pathways—plays a key role in the development and progression of IgAN and is closely associated with crescent formation. However, the original Oxford Classification cohort did not identify crescents as an independent predictor of eGFR decline or end-stage kidney disease (ESKD) (90). A study from Peking University First Hospital involving 100 IgAN patients (biopsied between 2004–2019) with varying crescent proportions revealed that patients with >50% crescent involvement had urinary levels of alternative and lectin pathway activation products 10- to 50-fold higher than those with fewer crescents. These elevated complement levels correlated with heavy proteinuria and reduced eGFR, suggesting a strong link between diffuse crescent formation and rapidly progressive renal failure (91). Studies by Haas and Shao also support that crescentic IgAN is associated with a poorer prognosis (92, 93). The 2017 update to the Oxford Classification (MEST-C ) incorporated a crescent score (C). Although an analysis by Trimarchi et al. suggested that patients with C1 score (<25% crescent involvement) still face poor renal outcomes without immunosuppressive therapy, and those with C2 score (≥25% crescents) remain at high risk even with such treatment (90), this does not negate the prognostic significance of crescentic lesions in IgAN. Rather, it highlights the need for early identification and tailored intervention in high-risk patients. Existing research on complement proteins in relation to the MEST-C classification has predominantly focused on C4d. Concurrently, urinary complement levels—as opposed to circulating complement—more accurately reflect local intrarenal complement activation. Among these, urinary C4d shows the most significant association, with its levels demonstrating a linear correlation with the proportion of crescentic glomeruli (r=0.562). This suggests that urinary C4d holds promise as a potential biomarker for monitoring crescent formation. The same study also found that the urinary C4d-to-creatinine ratio was associated with disease severity and progression in IgAN patients with crescents (94). The positivity rate for histological C4d staining increases in parallel with the percentage of crescentic glomeruli (91), further indicating that C4d could be a useful biomarker for monitoring crescent formation in this patient group. However, some studies have reported conflicting conclusions, showing no significant difference in the average C4d deposition score between crescent-positive (C≥1) and crescent-negative (C0) groups, or even observing a negative correlation between C4d positivity and crescent formation. This discrepancy may be attributed to factors such as small sample sizes, the focal nature of mesangial C4d deposition, low abundance of C4d following glomerular complement activation, and variations in detection techniques. Nevertheless, a study by Hiroe et al. involving 132 IgAN patients found that immunofluorescence scores for MASP1/3 and MASP2 were significantly higher in the C≥1 group compared to controls, providing further evidence for the involvement of lectin pathway activation in crescent formation (17).

Furthermore, the MEST-C scores (Mesangial hypercellularity, Endocapillary hypercellularity, Segmental glomerulosclerosis, Tubular atrophy/Interstitial fibrosis, Crescent) are well-established predictors of progression in IgAN. Dynamic histological evaluation through repeat renal biopsies, particularly noting changes in the T and C scores, can enhance the accuracy of predicting end-stage kidney disease (ESKD) (95). Therefore, elucidating the relationship between MEST-C lesions and the complement system may reveal potential therapeutic targets for IgAN.

#### 4.2.2 Lesions of small renal arteries

C4d deposition in IgAN is not confined to the glomeruli. Recent studies indicate that complement C4d is also associated with vascular lesions, particularly thrombotic microangiopathy (TMA)-like injuries (65). Concurrently, C4d deposition has been observed in intrarenal small arteries, where it is also linked to adverse renal outcomes in IgAN, suggesting its potential role as a biomarker for disease progression (65, 96). A study by Faria et al. involving 126 Portuguese adult IgAN patients found that 16.7% exhibited positive C4d immunohistochemical staining in small arteries, which correlated with hypertension, arterial intimal fibrosis, and chronic microangiopathy. The authors further hypothesized that abnormal activation of the MBL lectin pathway may contribute to intrarenal arteriolar injury (such as arteriolar hyalinosis and intimal fibrosis). Multivariate analysis indicated that the prognostic significance of arterial C4d staining for progressive renal dysfunction was even stronger than that of glomerular C4d deposition (96). However, this conclusion is derived from a single study in a specific population (126 Portuguese patients). Whether it applies to IgAN patients of different ethnicities and geographical regions requires validation through larger, multicenter, prospective cohort studies. However, it is important to note that current studies report that only 15–25% of IgAN cases involve the lectin pathway. In this subset of patients, the MBL pathway may contribute to intraglomerular arteriolar injury, which from a physiological perspective can be regarded as an expression of the “ phrasing of the pathological pentagram “ in such complex scenarios. We therefore hypothesize that the above pathological changes affect a specific patient population, and identifying these patients is crucial to avoid overtreatment with MASP-2 inhibitors, as excessive inhibition could lead to pathway deficiency. In 2022, a study from Anzhen Hospital involving 866 IgAN patients revealed that a subset of patients without hematuria (with or without hypertension) exhibited rapid renal function decline, characterized predominantly by intrarenal arteriolar pathology. Deposits of MBL, C4d, Factor H (FH), FHR5, C3c, and the membrane attack complex (MAC) were detected in the walls of these arterioles, suggesting that abnormal complement deposition may contribute to small vessel injury. However, since galactose-deficient IgA1 (Gd-IgA1) is rarely deposited in renal arterioles, and the intensity of C3c deposition in the arteriolar walls of the vascular injury group was significantly higher than in the glomerular deposition group, it appears that Gd-IgA1–mediated injury may be primarily localized to the glomeruli rather than the intrarenal arterioles (97). Furthermore, co-localization of complement C3c with the endothelial cell biomarker CD31 was observed in these arterioles, leading to the hypothesis that excessive complement activation may cause arteriolar damage via endothelial injury (96) . Thes e research findings suggest that renal small artery lesions in patients with IgA nephropathy may not be solely triggered by complement pathway activation.And robust evidence supporting standardized protocols for IgAN patients with concurrent TMA remains unavailable. Therefore, the specific mechanism by which the MBL pathway contributes to small arterial injury in IgAN remains to be further elucidated.

### 4.3 Body fluid biomarkers

IgAN often lacks obvious early clinical symptoms, leading to irreversible renal impairment in some patients by the time of diagnosis. Therefore, early detection and intervention are critical for improving outcomes (1). The absence of reliable non-invasive diagnostic alternatives necessitates continued reliance on renal biopsy as the gold standard for unequivocal diagnosis. Given the invasive nature of biopsy, there is an urgent need to explore non-invasive biomarkers that can reflect disease activity, aid in diagnosis, and predict prognosis.

Based on the “four-hit” hypothesis of IgAN, which involves serum Gd-IgA1 and its corresponding antibodies, multiple studies have reported that circulating levels of Gd-IgA1 are elevated in IgAN patients compared to those with other kidney diseases or healthy controls. These Gd-IgA1 levels have been correlated with histomorphological lesions and renal outcomes (98–100). However, a large systematic review and meta-analysis in 2024 found that the association between serum Gd-IgA1 levels and the risk of progressive renal function loss was inconsistent (101). Influenced by factors such as age, genetic background (102), and measurement methodologies, serum Gd-IgA1 lacks sufficient sensitivity and specificity (1). Therefore, current evidence does not support its use as a reliable biomarker for progressive disease.

As previously discussed, extensive evidence demonstrates co-deposition of MBL and IgA in Some of the IgAN patients, and glomerular deposits of MBL, MASPs (such as MASP1/3 and MASP2), and C4d correlate with disease severity. However, measuring these indicators still requires renal biopsy (103). Therefore, the focus has shifted to detecting relevant components in the blood and urine of IgAN patients to evaluate their potential as non-invasive diagnostic or prognostic biomarkers.

At the level of circulating components, both MBL and MASP-2 exhibit a “U-shaped” association with IgAN—where both excessively high and low levels are linked to markers of disease severity such as hematuria and proteinuria (24). Regarding serum C4d, although the earliest studies reported no correlation between changes in serum C4 and IgAN progression, systemic C4 activation has been observed in IgAN patients (evidenced by an elevated plasma C4d/C4 ratio in 28% of adult patients) along with alterations in serum C4BP (C4b-binding protein) levels. Some studies suggest that higher C4BP levels are associated with a poorer prognosis (104). However, these findings have not been consistently replicated, and the relationship between plasma C4d levels and the extent of glomerular C4d deposition requires further investigation. Additionally, changes in other lectin pathway components have been noted: compared to healthy controls, IgAN patients show elevated circulating levels of M-ficolin, L-ficolin, MASP-1, and MAp19, while MASP-3 levels are reduced. MASP-3 levels are significantly correlated with disease severity—patients with progressive IgAN have lower MASP-3 levels, which positively correlate with eGFR values (16). However, low MASP-3 levels are also observed in patients with systemic lupus erythematosus and nephritis (105) and are not specific to IgAN. Therefore, MASP-3 appears to have a distinct role not in the diagnosis of IgAN, but rather in the evaluation of disease severity.

IgAN is often asymptomatic in its early stages, with most cases being incidentally detected through routine urinalysis. The initial manifestations typically include hematuria and proteinuria, making urinary screening an effective method for early detection. To date, the only urinary biomarkers consistently associated with IgAN progression risk are eGFR and proteinuria (106). However, these parameters require long-term dynamic monitoring, lack sensitivity in identifying early subclinical injury, and do not directly reflect underlying renal pathological changes, limiting their utility in guiding treatment decisions. Therefore, there is a need to identify novel biomarkers that address these limitations. A study of 96 patients with biopsy-confirmed primary IgAN demonstrated that urinary C4d detection had 90% sensitivity and 73% specificity for identifying mesangial C4d deposition, while urinary MBL detection showed 83.9% sensitivity and 81.6% specificity for detecting mesangial MBL deposition (107). These findings suggest that measuring complement proteins in urine may serve as a non-invasive alternative for diagnosing and evaluating IgAN. But urinary MBL levels are not entirely dependent on proteinuria, so it may also be elevated in patients with low proteinuria. Currently, the role of urinary MBL as a biomarker for specific IgAN subtypes remains unclear and requires further investigation for confirmation (47). A cohort study of 168 IgAN patients with crescentic lesions found that urinary C4d levels or the urinary C4d-to-creatinine ratio were independent predictors of progressive renal failure (94). Nurmi et al. identified urinary complement-related proteins—u-PTX-3, u-MBL, and u-C4c—as potential biomarkers reflecting disease activity and chronic changes in both IgAN and IgA vasculitis with renal involvement (IgAVN) (56). A recent large-scale study analyzing urine samples collected at the time of biopsy from 508 patients with biopsy-confirmed IgAN demonstrated that urinary C4d levels are associated with disease progression. Sustained monitoring of urinary C4d may serve as an indicative marker for evaluating treatment response to lectin pathway inhibitory therapies (108). However, it is important to note that lectin pathway activation is neither universal in IgAN nor specific to it, as it is implicated in various other glomerular diseases. Therefore, large-scale, multicenter, prospective cohort studies are required to elucidate the distinct clinical significance of urinary C4d in IgAN.

## 5 Therapeutic strategies and advances in targeting the lectin pathway

Currently, there is no disease-specific therapy for IgAN. The KDIGO guidelines strongly recommend renin-angiotensin system (RAS) inhibitors, blood pressure control, cardiovascular risk management, and lifestyle modifications. The use of corticosteroids, non-steroidal immunosuppressive agents, and tonsillectomy remains controversial and is reserved as second-line treatment for specific cases (109). Over the past decade, research on the pathogenesis of IgAN has centered on dysregulated complement activation. This focus has driven the development of clinical trials for complement-targeted therapies. The lectin pathway, in particular, serves as the most direct link between IgAN-specific pathogenic mechanisms (Gd-IgA1-containing immune complexes) and downstream complement activation and inflammatory injury. As such, it has naturally become a central focus for both pathogenesis studies and targeted drug development.

Targeting the source of the “four-hit” process in IgA nephropathy, sibeprilimab—an investigational humanized IgG2 monoclonal antibody—specifically binds to and neutralizes APRIL (a proliferation-inducing ligand), blocking its interaction with receptors and reducing the production of pathogenic Gd-IgA1. Clinical data have shown significant reduction in proteinuria (up to 62% in Phase II studies). A Phase III trial (NCT05248646), which enrolled 470 participants, was initiated in March 2022 to further evaluate its efficacy and safet y (110). Notably, It not only serves as the initiating factor that activates the downstream lectin pathway of the complement system, but its own generation is also co-regulated by the complement alternative pathway and the B-cell regulatory axis. Evidence indicates that components of the alternative pathway (such as CFHR3) may be involved in the regulation of B cells (111). In patients with IgA nephropathy, serum BAFF levels correlate with clinical and pathological features (112). Furthermore, APRIL, in synergy with B lymphocytes, can induce elevated levels of Gd-IgA1 and is associated with progression to end-stage kidney disease (ESKD) (1, 113). Therefore, future targeted therapeutic strategies must comprehensively consider multiple factors, including BAFF, APRIL, and the alternative pathway.

It is noteworthy that inhibiting MASP-2 does not interfere with the classical complement pathway or compromise immune responses to infection (11), which may reduce the risk of potential immune dysregulation. Narsoplimab, a humanized monoclonal antibody targeting MASP-2, reduces complement-mediated glomerular inflammation.

Narsoplimab is a humanized monoclonal antibody targeting MASP-2 that mitigates complement-mediated glomerular inflammation. Although some positive signals were observed in a phase II study and its two sub-studies, the phase III trial enrolled 450 patients (225 per group), all of whom had biopsy-proven IgAN, proteinuria >1 g/day, and eGFR ≥30 mL/min/1.73 m². During the initial 12-week treatment period, patients were randomized to receive weekly intravenous narsoplimab or placebo, and the response assessment period (weeks 13–36) included monitoring of proteinuria response or an additional 6 weeks of blinded treatment. The primary endpoint was the change in proteinuria from baseline at 36 weeks. Ultimately, narsoplimab did not significantly reduce proteinuria compared to placebo, leading to the termination of the trial in 2023 (114). These findings indicate that in IgAN, simply inhibiting the lectin pathway may be insufficient to control all complement-mediated injury. This study represents a significant evaluation of the strategy of implementing single-pathway complement inhibition in an unselected population. Rather than concluding the exploration of the lectin pathway, it highlights the need to comprehensively consider the synergistic effects of multiple complement pathways in IgAN treatment and, more importantly, to move toward precision medicine for IgAN. SHR-2010 is a novel humanized IgG4 monoclonal antibody targeting MASP-2. A first randomized, double-blind Phase I study conducted by Pingping Lin et al. in 2025 demonstrated that a single dose of SHR-2010 was safe, well-tolerated, and effectively inhibited the lectin pathway in healthy adults. While its inhibitory potency was comparable to narsoplimab, it exhibited an extended duration of action, suggesting a potential for an improved overall pharmacological profile. A Phase II study evaluating the efficacy and safety of SHR-2010 in patients with primary IgA nephropathy is currently underway (NCT05847920) (115).

## 6 Conclusion

Although current research reports show that only 15-25% of IgAN patients involve the lectin pathway in the disease progression, and as discussed in the sections on genetics and MBL, the role of this pathway remains controversial, uncertain, and highly variable, it cannot be definitively asserted that the lectin pathway plays a role in IgA pathogenesis or whether it is activated by immune complexes. Meanwhile, the case of Narsoplimab further highlights the complexity of the pathogenesis of IgA nephropathy, as simply inhibiting MASP-2 failed to achieve the expected therapeutic efficacy. As discussed earlier, since C3b serves as a crucial bridge in both the alternative and lectin pathways, and MASP-3 cleaves pro-factor D into factor D, a critical link is established between the lectin and alternative pathways. Existing studies have shown that patients with MBL and C4d deposition exhibit more severe renal injury. The lectin pathway and the alternative pathway exhibit synergistic effects, collectively participating in the initiation and progression of the disease. The above shows that the activation of the lectin pathway plays a significant yet non-universal role in the disease progression and poor prognosis of a subset of IgAN patients. Its pathological significance is not necessarily that of a universal initiating factor but rather serves as an efficient “catalyst” and “amplifier.” Therefore, its potential role in disease development, its possibility as a therapeutic target, and its value in prognosis assessment cannot be denied, making it an indispensable part of IgAN research. By further studying the lectin pathway, it will not only help unveil the complex pathogenesis of IgAN but may also provide new insights and approaches for its diagnosis, treatment, and prognosis evaluation.

At the same time, we cannot overlook a major obstacle in the treatment of IgAN: the shortage of biomarkers for predicting outcomes and guiding therapy (116). While omics research has identified candidates, the translation of these findings into validated serum or urinary tests for clinical practice remains unrealized (117). This is also a key direction for our future research. Therefore standardization of their detection methods and elucidation of their precise clinical significance represent important objectives for future research. Furthermore, targeted therapies against the complement system represent a cutting-edge direction in IgAN drug development, holding promise for advancing personalized management of this disease.
